# Vascular hyperacetylation is associated with vascular smooth muscle dysfunction in a rat model of non-obese type 2 diabetes

**DOI:** 10.1186/s10020-022-00441-4

**Published:** 2022-03-08

**Authors:** Maria Alicia Carrillo-Sepulveda, Nicole Maddie, Christina Mary Johnson, Cameron Burke, Osina Lutz, Bamwa Yakoub, Benjamin Kramer, Dhandevi Persand

**Affiliations:** 1 Department of Biomedical Sciences, College of Osteopathic Medicine, New York Institute of Technology, Northern Blvd., Old Westbury, NY 11568 USA; 2 Department of Life Sciences, College of Arts and Sciences, New York Institute of Technology, Northern Blvd., Old Westbury, NY 11568 USA; 3 Department of General Surgery, Cleveland Clinic, Cleveland, OH 44195 USA

**Keywords:** Lysine acetylation, PCAF, Vascular dysfunction, Type 2 diabetes, High glucose, ROS, Vascular smooth muscle cells

## Abstract

**Background:**

Advanced type 2 diabetes mellitus (T2DM) accelerates vascular smooth muscle cell (VSMC) dysfunction which contributes to the development of vasculopathy, associated with the highest degree of morbidity of T2DM. Lysine acetylation, a post-translational modification (PTM), has been associated with metabolic diseases and its complications. Whether levels of global lysine acetylation are altered in vasculature from advanced T2DM remains undetermined. We hypothesized that VSMC undergoes dysregulation in advanced T2DM which is associated with vascular hyperacetylation.

**Methods:**

Aged male Goto Kakizaki (GK) rats, a non-obese murine model of T2DM, and age-matched male Wistar rats (control group) were used in this study. Thoracic aortas were isolated and examined for measurement of global levels of lysine acetylation, and vascular reactivity studies were conducted using a wire myograph. Direct arterial blood pressure was assessed by carotid catheterization. Cultured human VSMCs were used to investigate whether lysine acetylation participates in high glucose-induced reactive oxygen species (ROS), a crucial factor triggering diabetic vascular dysfunction.

**Results:**

The GK rats exhibited marked glucose intolerance as well as insulin resistance. Cardiovascular complications in GK rats were confirmed by elevated arterial blood pressure and reduced VSMC-dependent vasorelaxation. These complications were correlated with high levels of vascular global lysine acetylation. Human VSMC cultures incubated under high glucose conditions displayed elevated ROS levels and increased global lysine acetylation. Inhibition of hyperacetylation by garcinol, a lysine acetyltransferase and p300/CBP association factor (PCAF) inhibitor, reduced high glucose-induced ROS production in VSMC.

**Conclusion:**

This study provides evidence that vascular hyperacetylation is associated with VSMC dysfunction in advanced T2DM. Understanding lysine acetylation regulation in blood vessels from diabetics may provide insight into the mechanisms of diabetic vascular dysfunction, and opportunities for novel therapeutic approaches to treat diabetic vascular complications.

**Supplementary Information:**

The online version contains supplementary material available at 10.1186/s10020-022-00441-4.

## Background

With about 1.5 million individuals diagnosed each year, type 2 diabetes mellitus (T2DM) is becoming one of the most prevalent metabolic diseases affecting Americans (Prevention 2020). T2DM is a chronic metabolic disorder chiefly characterized by insulin resistance and high levels of blood glucose that ultimately lead to debilitating disorders of the vascular system (Fowler 2011; Sposito et al. 2018; Verges 2015). Vascular complications are the leading cause of death among diabetic patients, including coronary artery disease and stroke, at a rate two to four times higher than non-diabetics (Emerging Risk Factors et al. 2010).

While current advances in glucose-lowering medications have provided optimal blood glucose control in diabetic patients, these therapeutics do not abolish the progression of diabetic vascular complications (Holman et al. 2008; Action to Control Cardiovascular Risk in Diabetes Study et al. 2008). Clinical trials have revealed that tight glycemic control is not effective in resolving cardiovascular complications in both pre-T2DM, as defined by an HbA1C between 5.7% and 6.4% (Roberts et al. 2017; Tabak et al. 2012), and T2DM patients (Cai et al. 2020; Moodahadu et al. 2014; Despres et al. 1996; Caballero et al. 1999). Thus, the positive vascular effects of anti-hyperglycemic therapies during T2DM remains insufficient. Studies examining therapeutic strategies that can simultaneously lower glucose levels and promote vascular protection are urgently needed. To date, there is no treatment specifically targeting vascular complications in diabetes.

The prevalence of T2DM and vascular complications increases with age. Clinical and experimental studies have shown that endothelial dysfunction, characterized by impaired endothelium-dependent vasodilation, is detected in pre-T2DM and early stages of T2DM (Kazuyama et al. 2009; Su et al. 2008; Caballero et al. 1999). While endothelial dysfunction has been well-characterized in T2DM, VSMC dysfunction, which arises in advanced T2DM, remains less studied.

Recent evidence has increasingly shown that glucose triggers post-translational modifications (PTM) in diabetes which may play an important role in the pathogenesis of diabetic cardiovascular complications (Mellor et al. 2015; Di Tomo et al. 2021; Zhang et al. 2015). Among the PTMs, lysine acetylation, a reversible PTM that impacts protein activity, stability, and binding proteins, has recently been linked to cardiometabolic disorders such as obesity, metabolic syndrome, and cardiovascular diseases (Iyer et al. 2012; Hu et al. 2020). Advancements in high resolution mass spectrometry revealed that histones are not the sole target of lysine acetylation and that several non-histone proteins can also be acetylated and deacetylated, resulting in changes to their activity and expression (Choudhary et al. 2009; Sun et al. 2009; Chen et al. 2001; Glozak et al. 2005). Lysine N-ε-acetyltransferases, also known as KATs, catalyze these reactions by transferring an acetyl group from acetyl Co-A to lysine residues found on histones and non-histone proteins (Menzies et al. 2016). p300/CBP associated factor (PCAF) is a transcriptional co-activator with intrinsic acetyltransferase activity that promotes lysine acetylation (de Jong et al. 2017). Under proinflammatory conditions which affect the vasculature, PCAF is upregulated leading to an increase in inflammatory proteins and VSMC proliferation and migration (Qiu et al. 2019).

In the context of diabetes, high levels of global lysine acetylation were found in kidneys and correlated with diabetic nephropathy (Kosanam et al. 2014). Moreover, increased lysine acetylation of p66Shc, a regulator of ROS production, was reported to be associated with vascular oxidative stress and endothelial dysfunction in diabetic mice (Kumar et al. 2017). However, whether lysine acetylation-related mechanisms correlate with VSMC dysfunction in advanced T2DM has not yet been elucidated.

Our hypothesis, therefore, is that VSMC dysfunction in the advanced stage of diabetes is associated with vascular lysine acetylation. The aim of this study was to investigate whether advanced T2DM increases lysine acetylation in the vasculature, specifically in the VSMC, which in turn contributes to ROS production, a key component of diabetic vascular dysfunction.

## Methods

### Experimental model of T2DM

Goto Kakizaki (GK) rats, a polygenic non-obese and spontaneous model of T2DM, were utilized in this study. GK rats were obtained by repetition of selective breeding of glucose intolerant Wistar rats in order to develop a non-obese diabetic rodent model which develops T2DM without the compounding implications of obesity (Goto et al. 1976). Aged male 48-week-old GK rats (Diabetic Group) were obtained from Taconic Biosciences (Albany, NY, USA). The age of these rats was selected to reflect advanced T2DM (Hjortbak et al. 2018). Age-matched male Wistar rats (Control Group) were obtained from Charles River (New York, NY, USA). The rats were maintained on a 12-h light–dark cycle, receiving regular animal chow (Zeigler, Gardners, PA, USA) consisting of 5.0% fat, 48.7% carbohydrates (3.2% sucrose), and 24.1% protein (% total energy) and drinking water ad libitum. All experiments and protocols were conducted in accordance with the National Institutes of Health (NIH) Guidelines for the Care and Use of Laboratory Animals and approved by the New York Institute of Technology College of Osteopathic Medicine (NYIT-COM) Animal Care and Use Committee (Animals 2011).

### Metabolic parameters

Blood samples were collected at the experimental endpoint. After fasting for 8 h, blood samples were obtained from the tails of both diabetic and control groups to determine glucose levels by using an AimStrip Plus glucometer (Germaine Laboratories, San Antonio, TX, USA), triglycerides (TG) serum levels by using enzymatic commercial kits from Pointe Scientific (Canton, MI, USA), and non-esterified free fatty acid (NEFA) serum levels by using a colorimetric assay (NEFA C) from Wako Pure Chemical Industries (Richmond, VA, USA), according to the manufacturer’s instructions. Levels of glycated hemoglobin (HbA1C) were measured using a PTS Diagnostics A1C Now Multi-Test A1C System (Indianapolis, IN, USA).

### Oral glucose tolerance test (OGTT)

After 8 h of fasting, rats received 2 g/kg body weight of 20% glucose solution (Sigma, St. Louis, MO, USA) via oral gavage (Polce et al. 2018). Blood samples were obtained from tail veins of the rats immediately before (0 min) and 15, 30, 60 and 120 min after glucose solution administration. Blood glucose levels were obtained using an AimStrip Plus glucometer (Germaine Laboratories, San Antonio, TX, USA).

### Insulin resistance analysis

Insulin resistance was assessed by calculation of Triglyceride-glucose (TyG) index = Ln [fasting triglycerides (mg/dL) versus fasting blood glucose (mg/dL)/2)], a screening method for insulin resistance utilized in humans and rats (Ren et al. 2020; Gonzalez-Torres et al. 2015).

### Arterial blood pressure measurements

At the terminal experiments, direct arterial blood pressure was obtained, as previously described (Kramer et al. 2018). Rats were anesthetized with inhalation of 2.5% isoflurane in 100% oxygen flow, placed on a warming pad maintained at 37 °C and instrumented with a 1.9F SciSense pressure–volume catheter (Transonic SciScense Inc., London, ON, Canada) in the right carotid artery for direct blood pressure recording verified by pressure curves presented in the data acquisition system (Powerlab 4, ADInstruments; Bridge Amp, ML 110, Colorado Springs, CO, USA). Once the placement of the catheter was confirmed, isoflurane was reduced to 1.5% in 100% oxygen flow. After stabilization, blood pressure was continuously recorded for 30 min. The systolic and diastolic blood pressure were processed in a data acquisition system with Chart 7 software (ADInstruments, Colorado Springs, CO, USA).

### Vascular relaxation studies

VSMC-dependent relaxation in response to sodium nitroprusside (SNP) was evaluated.

At the time of experimental endpoint, thoracic aortas were quickly removed and carefully cleared of perivascular adipose tissue and adventitia in oxygenated Krebs buffer (130 mM NaCl, 14.9 mM NaHCO_3_, 4.7 mM KCl, 1.18 mM KH_2_PO_4_, 1.17 mM MgSO_4_-7H_2_O), 1.56 mM CaCl_2_-2H_2_O, 0.026 mM EDTA, 5.5 mM glucose, pH 7.4). Thoracic aortas were cut into rings (2 mm in length) and mounted on a Multi-Wire Myograph System 620 M (Danish Myo Technology, Aarhus, Denmark) for isometric tension recordings by a PowerLab 8/SP data acquisition system (ADInstruments Pty Ltd., Castle Hill, Australia). Aortic rings were then equilibrated in Krebs buffer for 30 min and gassed with 5% CO_2_ in 95% O_2_ at 37 °C (Carrillo-Sepulveda et al. 2015). Aortic rings were pre-contracted with phenylephrine (1 μM). After the phenylephrine-induced contraction reached a plateau, concentration–response curves for SNP (1 ηM to 0.01 mM) were performed.

### Quantification of vascular remodeling

At the time of the experimental endpoint, thoracic aortas were harvested, placed in Tissue-Tek O.C.T Compound (VWR catalog no. 25608–930; Sakura Finetek, CA, USA) and slowly snap frozen in an isopentenyl and dry ice emulsion bath, and stored at − 80 °C. Frozen sections were cut into 8 μM on cryostat at − 17 °C. Hematoxylin and eosin staining (Sigma Aldrich, St. Louis, MO, USA) was performed on aortic cross sections. Samples were then analyzed for vascular remodeling using Image J software according to established protocols (Gomez-Roso et al. 2009; Maia et al. 2014). Image J analysis included measures of aortic wall thickness, cross sectional area (CSA), wall thickness per lumen ratio (Wm/L), and lumen diameter, as previously described (Carrillo-Sepulveda et al. 2019). Slides were digitized using an Olympus BX53 fluorescent microscope (Olympus America, Inc., Center Valley, MA, USA), high-resolution regular light digital images were captured under high-power magnifications (× 4) using an Olympus DP72 (Olympus America, Inc., Center Valley, MA, USA).

### Western blot analysis

Protein content was determined in the supernatant of aortic extracts using a commercial BCA kit (ThermoScientific, Rockford, IL, USA), according to the instructions. Equivalent amounts of protein (20 μg per lane) from aortas from each experimental group were loaded and separated by 10% sodium dodecyl sulfate–polyacrylamide gel electrophoresis (SDS-PAGE) and transferred to PVDF membranes (Thermo Fisher Scientific Inc., Rockford, IL, USA), as previously described (Carrillo-Sepulveda et al. 2015). Membranes were blocked with 5% non-fat milk solution in Tris-buffered saline with 0.1% tween (TBST) for 1 h at room temperature, and incubated overnight at 4 °C with the following specific primary antibodies: Acetylated Lysine (1:500, cat.n.9441, Cell Signaling Technology, Danvers, MA, USA) and PCAF (1:500, sc-13124, Santa Cruz Biotechnology, Santa Cruz, CA, USA). As loading controls, β tubulin (1:20.000, cat.n.86298, Cell Signaling Technology, Danvers, MA, USA) and β actin (1:20.000, cat.n.4967, Cell Signaling Technology, Danvers, MA, USA) were utilized. Following incubation with secondary antibodies, bands were detected with the enhanced chemiluminescence system (Amersham Biosciences, Waltham, MA, USA). Immunoblots were quantified using Image J software, distributed by the NIH, and presented as a percent (%) of control.

### Aortic VSMCs cultures

Primary aortic VSMC cultures were obtained by enzymatic digestion of the thoracic aortas of male Wistar and GK rats, as previously described (Carrillo-Sepulveda et al. 2015). Isolated aortic VSMCs were placed in a culture dish and maintained in Dulbecco Modified Eagle’s Medium (DMEM) containing 10% fetal bovine serum (FBS) and antibiotics in a humidified incubator at 37℃, 5% CO_2_ and atmospheric O_2_. After confluence, VSMCs exhibited the typical “hill and valley” growth morphology and were confirmed positive (> 95%) for smooth muscle α-actin. Cells at early passage (Passage 1) were used for phenotypic characterization. Male human aortic VSMC (hVSMC) were obtained from the American Type Culture Collection (Manassas, VA, USA). hVSMC were grown in DMEM supplemented with antibiotics and maintained at 37 °C in a 5% CO_2_ incubator. After reaching confluence, hVSMC were incubated in serum-free medium overnight to reach a quiescence state (Kramer et al. 2018). After this period, cells were incubated with high glucose (HG, 25 mM) for 12 h (Carrillo-Sepulveda et al. 2015). In some experiments, cells were pre-incubated with 15 μM of garcinol, a PCAF inhibitor (cat.n. BML-GR343-0050, Enzo Life Sciences, Farmingdale, NY, USA), or vehicle control for 30 min. The concentration of garcinol used in this study was chosen based on previously published data (Bastiaansen et al. 2013; de Jong et al. 2017). The effect of garcinol on cell viability was assessed by using MTT assay (Zhou et al. 2016) (See Additional file 1: Material and Methods). Control hVSMC were maintained in normal glucose (NG, 5 mM). To rule out possible influence of osmotic stress, cells were also incubated in 25 mM mannitol (Rozentsvit et al. 2017).

### Measurement of reactive oxygen species (ROS).

ROS levels were detected in hVSMC from either the NG or HG group and in primary aortic VSMC from GK rats with 25 μM dihydroethidium (DHE; Sigma-Aldrich, St. Louis, MO, USA), as previously described (Kramer et al. 2018). hVSMC and primary aortic VSMC from GK rats stimulated with HG in the presence and absence of 15 μM garcinol were incubated with DHE in the medium for 20 min, and ROS levels were then detected. Fluorescence from DHE was detected using an Olympus DP73 fluorescence microscope with a Nikon digital camera. Quantitative analysis was performed to detect changes in fluorescence in hVSMC using Image J software (NIH).

### Statistical analysis

Results are expressed as means ± SEM and analyzed with a student’s two-tailed t-test or ANOVA, comparing the controls and GK groups, and the NG, HG, Garcinol and HG + Garcinol groups. Vascular reactivity contractions were recorded as changes in the displacement (mN) from baseline. Concentration–response curves were log-transformed, normalized to percent maximal response, and fitted using a nonlinear interactive fit (Graph Pad Prism 4.0; GraphPad Software Inc., San Diego, CA, USA). Emax (maximum relaxation response). pD2 (-log of the half-maximal effective concentration [EC_50_]). P-values less than 0.05 were considered significant.

## Results

### Altered metabolic profile in GK rats

**Fig. 1:**
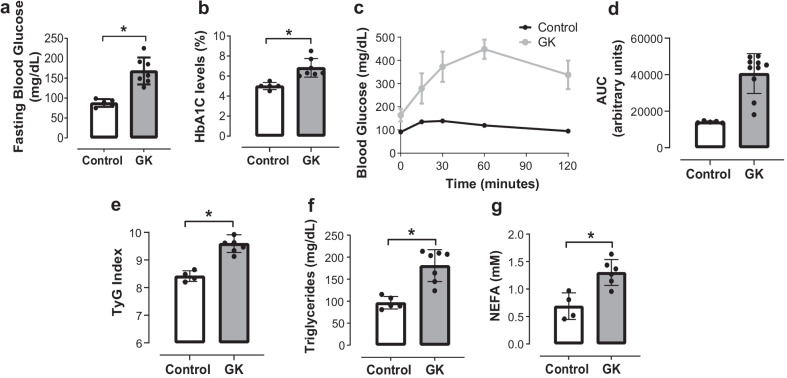
Altered glucose metabolism, insulin sensitivity and lipid profile from GK rats in advanced T2DM. **a** Fasting blood glucose (mg/dL), **b** HbA1C levels (%), **c** Oral glucose tolerance test (oGTT) performed during 120 min after administration of glucose (2 g/kg body weight), **d** Total blood glucose accumulation reported as area under the curve (AUC), **e** TyG Index (insulin sensitivity marker), **f** Fasting triglycerides (mg/dL), **g** Non-esterified fatty acids (NEFA) (mM) of male GK rats. *p < 0.05 vs. control, n = 5–7 per group. Values are means ± SEM

Body weight was lower in GK rats (382.83 g ± 18.77 g, p < 0.05) in comparison with age-matched Wistar rats (545.16 g ± 39.33 g). Altered glucose metabolism has been well-documented in young and adult GK rats (Palygin et al. 2019). Here we determined glucose metabolism in aged 48-week-old GK rats. High fasting blood glucose (Fig. 1a) and HbA1C above 6.5% (Fig. 1b) were identified in aged GK rats, confirming advanced T2DM stage. Significant glucose intolerance (Fig. 1c, d) in the GK group was accompanied by insulin resistance, as confirmed by increased TyG index (Fig. 1e). Lipid profile was altered in the aged GK rats as evidenced by elevated fasting triglycerides (Fig. 1f) and NEFA levels (Fig. 1g).

### Elevated blood pressure and aortic VSMC dysfunction in GK rats

**Fig. 2:**
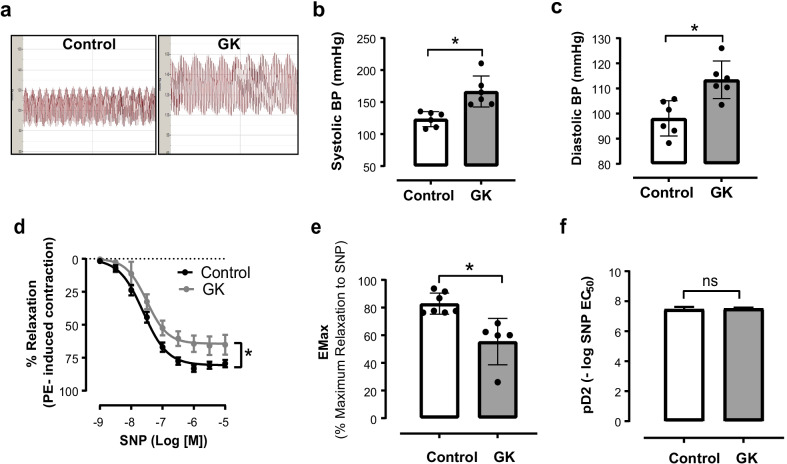
Increased arterial blood pressure, and impaired VSMC-dependent relaxation from GK rats in advanced T2DM.** a** Representative tracing of direct arterial blood pressure (BP) measurement via carotid catheterization. **b** Systolic BP (mmHg) and **c** Diastolic BP (mmHg)**. d** Cumulative concentration–response curves to Sodium Nitroprusside (SNP) in aortas from GK rats (gray circles) in comparison to aortas from age-matched male Wistar rats (black circles). Each point represents the maximal response to each concentration of SNP, **e** Maximal relaxation response (Emax) to SNP, **f** pD2 for SNP curve. *p < 0.05 vs. control, n = 6 per group. Values are means ± SEM

GK rats have been shown to exhibit elevated arterial blood pressure from a young age (Ma et al. 2017; Cui et al. 2014). Likewise, aged GK rats continue to show significant high systolic and diastolic blood pressure (Fig. 2a–c). To determine whether high blood pressure is accompanied by VSMC dysfunction, VSMC-dependent relaxation was assessed by using wire myograph. As shown in the Fig. 2d, VSMC-dependent relaxation was significantly reduced in the GK group. While maximum relaxation response (Emax) to SNP was reduced in the GK group (Fig. 2e), no differences in the sensitivity to SNP were observed between the experimental groups (Fig. 2f).

### Aortic remodeling in GK rats

**Fig. 3:**
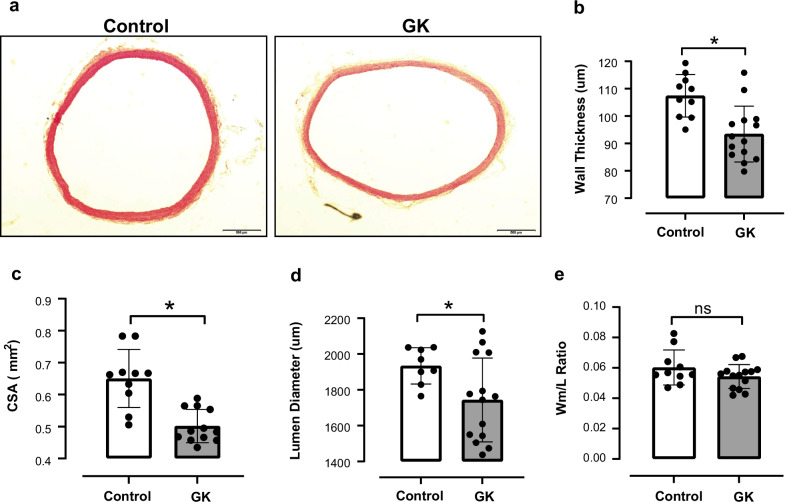
Vascular remodeling in aortas from GK rats in advanced T2DM.** a** Representative photomicrographs of the aortic cross-sections from the control and GK groups stained with H&E. Scale bar: 500 μm. **b** Wall thickness (Wm) (μm), **c** Cross-Sectional Area (CSA) (mm^2^), **d** Lumen diameter (μm), and **e** Wall-to-lumen (W_m_/L) ratio were obtained using Image J analysis software. *p < 0.05 vs. control, n = 6–10 per group. Values are means ± SEM

Aortic remodeling has been documented in young GK rats (Chettimada et al. 2014). However, less is known about aortic remodeling in aged GK rats. We found that aged GK rats exhibited vascular remodeling (Fig. 3a), as confirmed by reduced wall thickness (Fig. 3b), decreased CSA (Fig. 3c) and decreased lumen diameter (Fig. 3d). There was no changes in the Wm/L ratio (Fig. 3e) between the control and GK groups.

### Hyperacetylation in aortas from GK rats

**Fig. 4:**
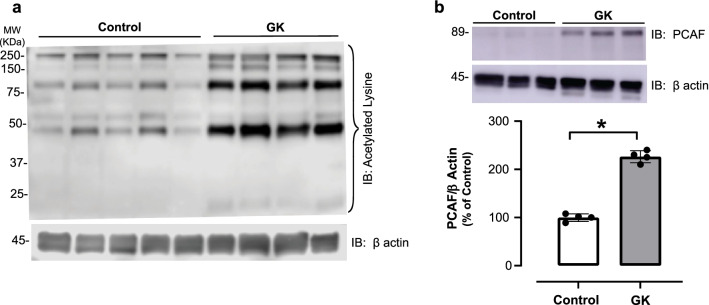
Lysine acetylation and PCAF expression is increased in aortas from GK rats in advanced T2DM. **a** Global lysine acetylation was detected by using a specific antibody for acetylated lysine residue. β actin used as internal control. **b** Representative immunoblotting for PCAF (top panel). β actin used as internal control. Bar graphs are means ± SEM of four independent experiments as determined from densitometry relative to β actin. *p < 0.05 vs. control, n = 4–5 per group

To address whether advanced T2DM causes vascular hyperacetylation, global lysine acetylation and PCAF levels were assessed in thoracic aortas from aged GK rats by western blot analysis. As shown in Fig. 4a, aortas from aged GK rats exhibited increased global lysine acetylation levels in comparison to the control Wistar rats. PCAF expression was also significantly elevated in thoracic aortas from aged GK rats (Fig. 4b).

### Increased lysine acetylation in aortic VSMCs

**Fig. 5:**
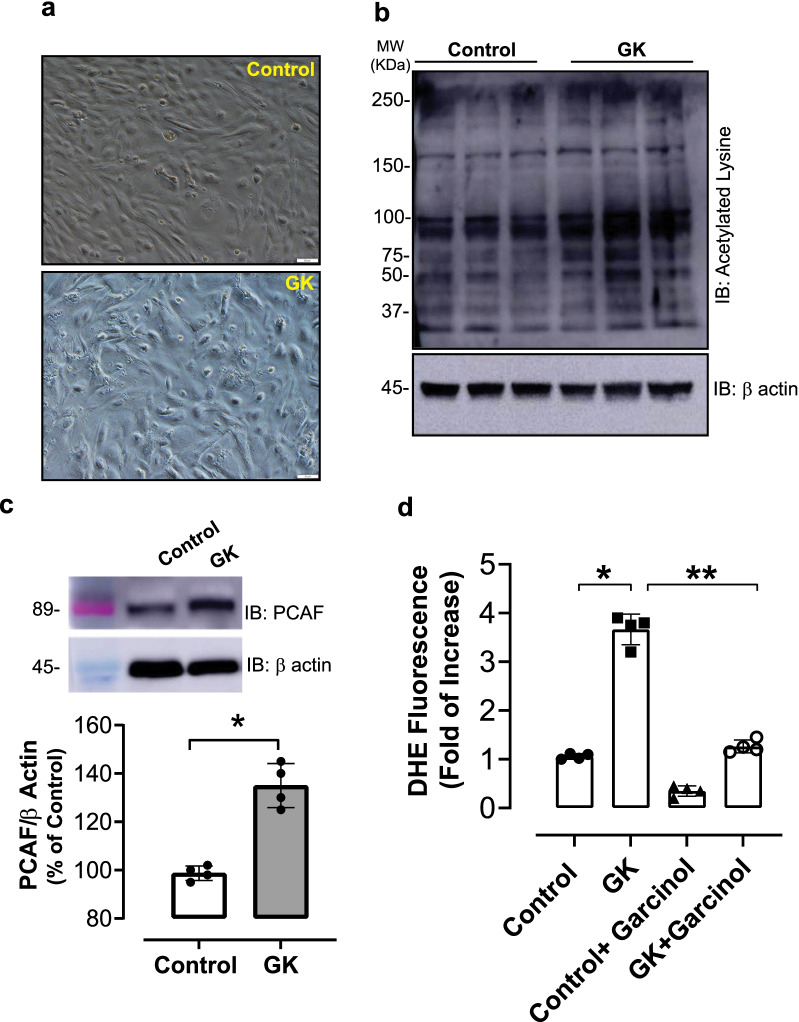
Lysine acetylation and PCAF expression is increased and ROS levels is elevated in aortic-VSMC from GK rats in advanced T2DM. Aortic VSMCs were isolated from Wistar and GK rats and maintained in primary cultures. **a** Morphology of aortic VSMCs cultures in passage 1. **b** Global lysine acetylation was detected by using a specific antibody for acetylated lysine residue. β actin used as internal control. **c** Representative immunoblotting for PCAF (top panel). β tubulin used as internal control. **d** ROS levels detected by DHE fluorescence in each experimental group. Quantification of DHE staining was determined through fluorescent intensity in each cell by pixel intensity of the cell. Bar graphs are means ± SEM of four independent experiments as determined from densitometry relative to internal control. *p < 0.05 vs. control

To specifically assess aortic VSMC lysine acetylation status, VSMCs were isolated from thoracic aortas from aged GK rats and maintained in primary cultures. While aortic VSMCs from age-matched Wistar exhibited organized spindle-shape arrangement in culture, aortic VSMC from aged GK rats exhibited a disorganized cellular arrangement, losing its native spindle-shape (Fig. 5a). Aortic VSMC from aged GK rats also exhibited elevated lysine acetylation levels (Fig. 5b), PCAF (Fig. 5c) expression, and elevated ROS levels which was reduced in presence of 15 μM of garcinol (Fig. 5d).

### High glucose increases lysine acetylation in human VSMCs

**Fig. 6:**
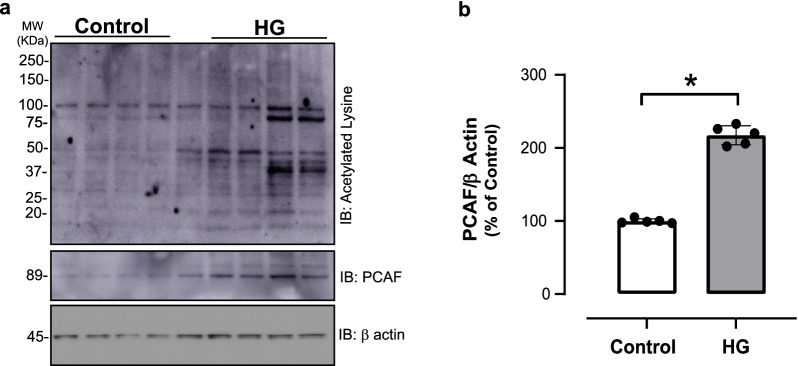
High glucose increases levels of lysine acetylation and PCAF expression in primary human VSMCs cultures. Human VSMCs (hVSMC) were stimulated with 25 mM high glucose (HG) for 12 h. **a** Global lysine acetylation was detected by using a specific antibody for acetylated lysine residue. β actin used as internal control. **b** Bar graphs are means ± SEM for PCAF expression as determined from densitometry relative to internal control. β tubulin used as internal control. *p < 0.05 vs. control, n = 5 per group. Molecular weight (MW)

T2DM is characterized by hyperglycemia associated with insulin resistance (Rizza 2010). To determine whether high levels of glucose can directly alter lysine acetylation levels, male human aortic VSMCs were stimulated with HG. As shown in the Fig. 6a, HG marked increased lysine acetylation levels in human aortic VSMCs. Moreover, increased PCAF expression was detected in human aortic VSMCs stimulated with HG (Fig. 6b).

### Inhibition of hyperacetylation reduces HG-induced ROS production in hVSMCs

**Fig. 7:**
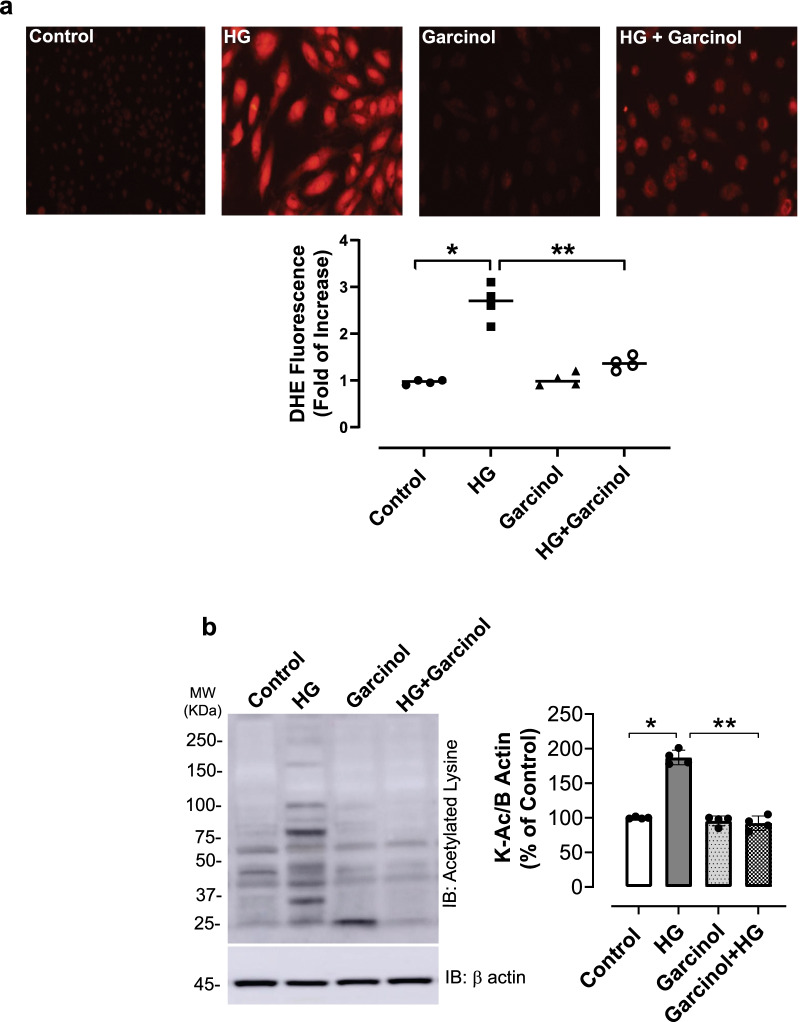
Garcinol reduces HG-induced ROS production in hVSMC. Quiescent hVSMC were pretreated with 15 µM garcinol, followed by stimulation with 25 mM HG or 5 mM NG for 60 min. **a** ROS production was detected using DHE staining. Representative photomicrographs depicting DHE fluorescence in each experimental group. Quantification of DHE staining was determined through fluorescent intensity in each cell by pixel intensity of the cell. **b** Global lysine acetylation was detected by western blot. β actin used as internal control. Results represent means ± SEM. n = 4 per group, *p < 0.05 vs. controls, **p < 0.05 vs. HG group

HG increases ROS levels in VSMC (Fiorentino et al. 2013). PCAF promotes acetylation of proteins involved in vascular dysfunction (de Jong et al. 2017). Here we hypothesized that HG- induced ROS generation in VSMC occurs via hyperacetylation-related mechanisms. As shown in Fig. 7A, HG significantly increased ROS levels in hVSMCs. This effect was attenuated in the presence of 15 μM garcinol, a concentration that inhibits lysine acetylation (Fig. 7B). This concentration of garcinol did not affect VSMC viability (See Additional file 2: Fig. S7A) nor inhibit other acetyltransferases, such as CREB-binding protein (CBP) (See Additional file 3: Fig. S7B).

## Discussion

The main findings of the present study are that in advanced T2DM, there is (1) impaired VSMC-dependent relaxation and (2) increased vascular lysine acetylation and PCAF expression in association with, (3) augmented ROS production in VSMC. Together, these results show that advanced T2DM negatively impacts VSMC function resulting in heightened impaired vasodilation, and imply vascular lysine acetylation as a potential factor involved in diabetic vascular dysfunction.

Patients with T2DM are two times more likely to develop cardiovascular complications. As many as 80% of diabetic patients manifest some macrovascular complication during the disease (Buse et al. 2007). Impaired vasodilation is a common vascular outcome in patients with T2DM (Sena et al. 2011; Kazuyama et al. 2009), which ultimately leads to cardiovascular complications such as hypertension and coronary artery disease (Su et al. 2008). It is well-established that endothelial dysfunction is an initial factor causing impairment of vasodilation in early stages of T2DM (Shi and Vanhoutte 2017; Sena et al. 2011). However, less is known about the temporal contribution of VSMC dysfunction in the reduced vasodilation mainly in the advanced stage of T2DM.

In this study, we found that GK rats in the advanced stage of T2DM display reduced vasodilation because of VSMC dysfunction. This data is in accordance with previous studies showing that GK rats exhibit VSMC dysfunction at 36 and 70 weeks of age (Kazuyama et al. 2009; Kobayashi et al. 2004).

Vascular dysfunction is one of the central factors contributing to the development of hypertension in diabetics (Sowers et al. 2001; Petrie et al. 2018; King 1996). While endothelial dysfunction has been implicated in hypertension in the early stages of T2DM (Regensteiner et al. 2005), results from the current study suggest that VSMC dysfunction may additionally be contributing to sustained high systolic and diastolic blood pressure present in advanced T2DM. Clinical studies have demonstrated that hypertension is two times more likely in diabetic patients and increases the risk of serious cardiovascular events (Simonson 1988; Gaede et al. 2003; Paula et al. 2015). While our findings demonstrate an association between advanced T2DM and hypertension in male rats, further studies using resistance arteries are warranted to address mechanisms of hypertension in advanced T2DM. Moreover, including a female cohort is necessary to address sex differences in the prevalence of T2DM and its related hypertension (Regensteiner et al. 2015).

An important factor to take into consideration in diabetic vascular complications is dyslipidemia, a common finding in advanced T2DM, which negatively affects vascular integrity leading to premature atherosclerotic disease (Schofield et al. 2016). Our present results show that GK rats, a non-obese model of T2DM with normal lipid profile, develop dyslipidemia in advanced stage of T2DM, as evidenced by elevated levels of triglycerides and NEFA. Thus, together with VSMC dysfunction, dyslipidemia can further potentiate vascular dysfunction in advanced T2DM, supporting increased cardiovascular events in diabetics (Wannamethee et al. 2011; Zoungas et al. 2014).

Clinical and experimental studies have reported arterial remodeling in T2DM (Sachidanandam et al. 2009; Elgebaly et al. 2010; Faries et al. 2001). In early stages of T2DM in young GK rats, wall thickness is increased or shows no changes, and displays a hypertrophic remodeling characterized by wall hypertrophy (Chettimada et al. 2014; Sachidanandam et al. 2009; Elgebaly et al. 2010) and increase in the CSA by either cell hyperplasia or hypertrophy (Edwards et al. 2020; Chettimada et al. 2014). In contrast, our results show that aortas from GK rats in the advanced stage of T2DM exhibited reduced wall thickness and CSA, which are indicative of hypotrophic vascular remodeling (Baleanu et al. 2009). The lumen diameter significantly decreased to a degree that is proportional to the wall thickness, as evidenced by the maintained Wm/L ratio. The decreased lumen diameter characterizes these findings as inward changes (Edwards et al. 2020). Therefore, the vascular remodeling in this model of advanced T2DM is categorized as an inward hypotrophic remodeling (Edwards et al. 2020; Maia et al. 2014). This type of remodeling could be due to vessel wall atrophy from a decrease in cell numbers or a decrease in cell size (Briones et al. 2006). In fact, it has been shown that hyperacetylation of specific proteins, such as Forkhead Box O (FoxO), mediates atrophy process (Bertaggia et al. 2012). Acetylation and other PTMs have been implicated in the remodeling of VSMCs in previous studies (Sun et al. 2017; Soe et al. 2014). While studies have provided evidence that hyperacetylation contributes to muscle wasting and muscle atrophy, these results remain contradictory, suggesting that more studies investigating the role of acetylation and deacetylation in muscle cells are needed (Alamdari et al. 2013). Sun, et al., reported that in a model of spontaneous hypertension, inhibition of histone acetyltransferases prevented VSMC proliferation and subsequent vascular remodeling (Sun et al. 2017). Of note, the rats used in that study were younger than those used in the current study and represented a model of spontaneous hypertension that is uncomplicated by age and diabetes (Sun et al. 2017). Our results shed light on a potential change in vascular remodeling from hypertrophic to hypotrophic remodeling with advanced T2DM in association with lysine hyperacetylation. Thus, our results suggest that hyperacetylation is a potential candidate contributing to vascular remodeling in T2DM, indicating that methods to modulate protein acetylation is an important field for continued research aimed to preventing and treating VSMC dysfunction.

Lysine acetylation is an important PTM that affects protein activity, stability, and binding properties (Li et al. 2020). Deacetylation and/or hyperacetylation of lysine has been linked with metabolic disorders including obesity, diabetes, and metabolic syndrome (Iyer et al. 2012). Specifically, studies in the field of diabetes have demonstrated that lysine acetylation levels are elevated in the heart, kidneys, and vasculature, suggesting its potential role in diabetic cardiovascular disease and nephropathy (Kosanam et al. 2014; Vazquez et al. 2015; Kumar et al. 2017). Our group has recently identified that deacetylation of peroxisome proliferator-activated receptor γ (PPARγ), a target factor in diabetes treatment, possesses an endothelial-protective effect (Liu et al. 2020). While previous reports have linked hyperacetylation to endothelial dysfunction in diabetes, our present study revealed that hyperacetylation is also associated with dysfunction of the VSMC layer, a component of the vasculature that is also affected as diabetes progresses (Kumar et al. 2017; Kazuyama et al. 2009). Additionally, we found that PCAF is upregulated in aortas from GK rats. It has been previously reported that inhibition of PCAF leads to reduction of acetylation and a corresponding decrease in inflammatory molecules (Huang et al. 2015; Malek et al. 2019). Our data show an association between increased global lysine acetylation and increased PCAF expression in aortas, specifically in VSMC, of aged GK rats. These results support the linkage between hyperacetylation and VSMC dysfunction in advanced T2DM that may be occurring via an imbalance between acetyltransferase and deacetylase activity, in which acetyltransferases, such as PCAF, is upregulated. While endothelial dysfunction arises in early stages of diabetes, VSMC dysfunction appears during advanced stages of T2DM. Increased lysine acetylation may play an integral role in both endothelial- and VSMC-dependent dysfunction.

**Fig. 8:**
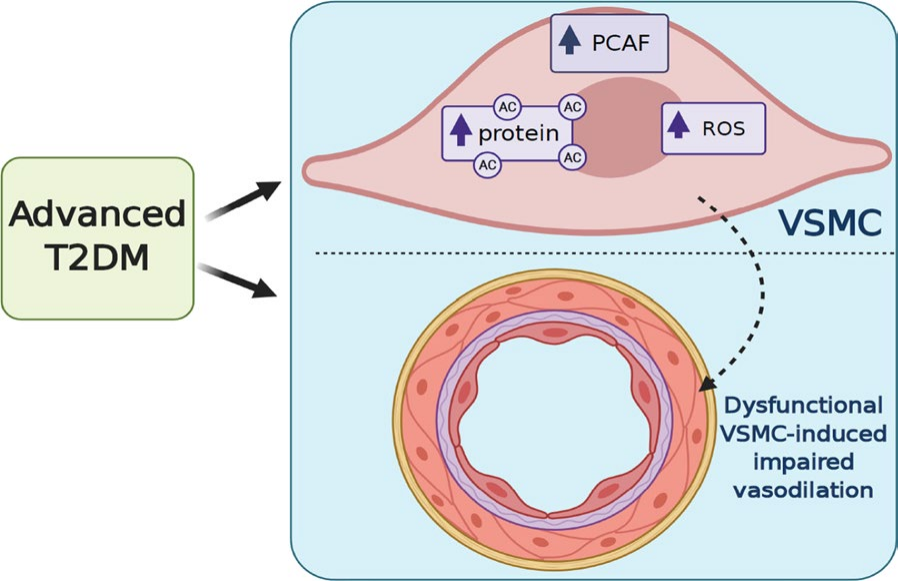
Schematic summary of the present study. Advanced T2DM leads to increased global lysine acetylation (AC) and PCAF expression in association with elevated ROS formation in VSMCs, which may be contributing to dysfunctional VSMC-induced impaired vasodilation. Created with BioRender.com

Vascular ROS production and its associated oxidative stress has been recognized as a key contributor to vascular dysfunction in diabetes (Di Fulvio et al. 2014; Pandolfi et al. 2003). To date, even though anti-oxidants have shown promising results in experimental studies, those results have not translated to clinical studies in humans (Hu and Liu 2016). In fact, clinical studies have identified that antioxidants, such as resveratrol and vitamin C, can reduce ROS levels. However, these antioxidants have not proven to be an effective deterrent of damage to the vasculature (Bo et al. 2016; Darko et al. 2002). Thus, a therapeutic antioxidant approach is urgently needed to treat vascular complications in diabetes. Recently, hyperacetylation of key proteins has been linked with elevated ROS and oxidative stress to the vasculature. Specifically, a recent study demonstrated that in diabetes, there is lysine acetylation of p66Shc, a master regulator of ROS. This leads to increased ROS-related endothelial dysfunction that is prevented by deacetylation of p66Shc (Kumar et al. 2017). Moreover, a recent study showed that diabetic conditions negatively regulates antioxidant properties in endothelial cells and this effect was associated with acetylation of p53 and increased expression of p300 (Di Tomo et al. 2021) These findings are in accordance with our results showing that elevated lysine acetylation is associated with dysfunctional VSMCs in diabetes. Given these findings, an approach that may effectively protect the vasculature from ROS insult is a treatment that prevents vascular hyperacetylation. Our data showed increased levels of the PCAF acetyltransferase in aortas from GK rats and in VSMC treated with HG in association with augmented lysine acetylation and ROS production. Treatment of VSMC with garcinol, a PCAF inhibitor, significantly reduced ROS formation, suggesting that inhibition of hyperacetylation-induced PCAF may be a potential treatment to protect the vasculature and prevent long term cardiovascular complications in diabetics. Worldwide increasing T2DM prevalence demands improvements in drug design and targeting PTMs correlated with diabetic complications poses a promising approach for treating T2DM and its vascular complications (Frkic et al. 2021; Stelmaszyk et al. 2021). In summary, findings from this study suggest that in advanced T2DM, PCAF is upregulated leading to increased vascular lysine acetylation and subsequent ROS production in VSMC, resulting in VSMC-related impaired vasodilation in advanced T2DM (Fig. 8).

### Study limitations

A limitation of this study is that we have not identified a specific protein(s) target that is hyperacetylated in advanced T2DM. Our results clearly show an increase in global lysine acetylation; however, future studies are required to determine the precise protein that is hyperacetylated. Our results suggest that postulated protein acetylation of interest is involved in the ROS production pathway as garcinol successfully inhibited lysine acetylation and decreased ROS production. Another limitation is the timeline of the development of VSMC-dependent dysfunction. There is controversial data reporting VSMC dysfunction in GK rats at early stages of T2DM (Kazuyama et al. 2009; Kobayashi et al. 2004). Future studies are required to precisely determine the timeline of VSMC dysfunction development. Lastly, garcinol treatment was assessed in an in vitro setting. Further exploration into the use of therapeutics, such as garcinol, in an in vivo setting is required.

## Conclusions

In summary, hyperacetylation of lysine residues in VSMCs is associated with impaired VSMC-dependent vasodilation in advanced T2DM. Additionally, reducing lysine acetylation by inhibiting acetyltransferases, such as PCAF, show a promising area for potential therapeutic treatments of vascular dysfunction in diabetic patients.

## Supplementary Information

**Additional file 1:** Material and Methods—MTT Assay Cell Viability.

**Additional file 2: Figure S7A. **Viability in VSMCs treated with garcinol.

**Additional file 3: Figure S7B. **CBP expression in VSMCs treated with HG and garcinol.

## Data Availability

The datasets used and/or analyzed are available from the corresponding author upon reasonable request.

## Abbreviations

T2DM: Type II diabetes mellitus
VSMC: Vascular smooth muscle cell
GK: Goto Kakizaki
ROS: Reactive oxygen species
PCAF: P300/CBP association factor
PTM: Post-translational modification
TG: Triglycerides
NEFA: Non-esterified free fatty acids
OGTT: Oral glucose tolerance test
TyG: Triglyceride-glucose index
SDS-PAGE: Sodium dodecyl sulfate–polyacrylamide gel electrophoresis
TBST: Tris-buffered saline with 0.1% tween
DMEM: Dulbecco modified Eagle’s medium
FBS: Fetal bovine serum
hVSMC: Human vascular smooth muscle cell
NG: Normal glucose
HG: High glucose
DHE: Dihydroethidium
SNP: Sodium nitroprusside
PPARγ: Peroxisome proliferator-activated receptor γ
