# Supplementary material for: Vascular hyperacetylation is associated with vascular smooth muscle dysfunction in a rat model of non-obese type 2 diabetes
Source: Mol Med. 2022 Mar 8;28:30. doi: 10.1186/s10020-022-00441-4 (PMC8902773; doi:10.1186/s10020-022-00441-4)
Supplement: Supplementary file 1 — Additional file 1: Material and Methods—MTT Assay Cell Viability. [file 10020_2022_441_MOESM1_ESM.docx]

**Additional File 1:** Supplemental Materials and Methods

MTT Assay Cell Viability

Prior to viability assay experiment, VSMCs were made quiescent by serum starvation for 24h. After that, VSMCs were seeded at a density of 3×10^3^ cells/well in 96-well plates and incubated with 15 μM garcinol for 12h. After the incubation period, 10μl of MTT (tetrazolium salt 3-(4,5-dimethylthiazol-2-yl)-2,5-diphenyltetrazolium bromide) labeling reagent (final concentration 0.5mg/mL to each well) was added to each well and incubated for 4h at 37°C. Light absorbance (570 nm) was measured with microplate reader (BioTek Cytation 1, Winooski, VT, USA) and quantified as per the manufacture’s protocol (Sigma Aldrich, St. Louis, MO, USA). Cell viability was expressed as a percentage relative to control in the absence of garcinol.
