# Supplementary material for: Vascular hyperacetylation is associated with vascular smooth muscle dysfunction in a rat model of non-obese type 2 diabetes
Source: Mol Med. 2022 Mar 8;28:30. doi: 10.1186/s10020-022-00441-4 (PMC8902773; doi:10.1186/s10020-022-00441-4)
Supplement: Supplementary file 2 — Additional file 2: Figure S7A. Viability in VSMCs treated with garcinol. [file 10020_2022_441_MOESM2_ESM.docx]

**Additional File 2:** Fig. S7-A


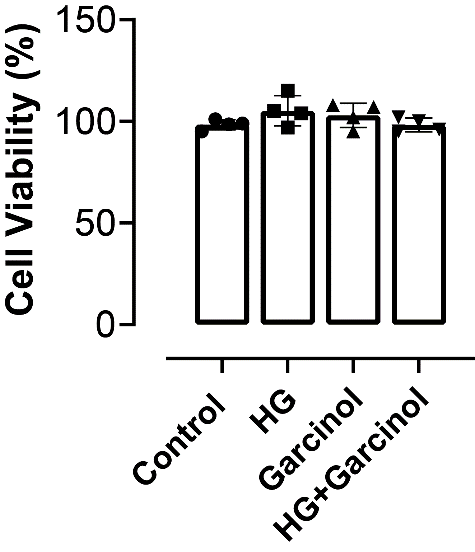


Fig. S7-A. Garcinol did not affect VSMCs viability. Human VSMCs (hVSMC) were pre-incubated with 15μM garcinol for 30 min followed by stimulation with 25 mM high glucose (HG) for 12h. VSMCs viability was detected by using MTT assay. Bar graphs are means ± SEM of four independent experiments.
