# Supplementary material for: Vascular hyperacetylation is associated with vascular smooth muscle dysfunction in a rat model of non-obese type 2 diabetes
Source: Mol Med. 2022 Mar 8;28:30. doi: 10.1186/s10020-022-00441-4 (PMC8902773; doi:10.1186/s10020-022-00441-4)
Supplement: Supplementary file 3 — Additional file 3: Figure S7B. CBP expression in VSMCs treated with HG and garcinol. [file 10020_2022_441_MOESM3_ESM.docx]

**Additional File 3:** Fig. S7-B


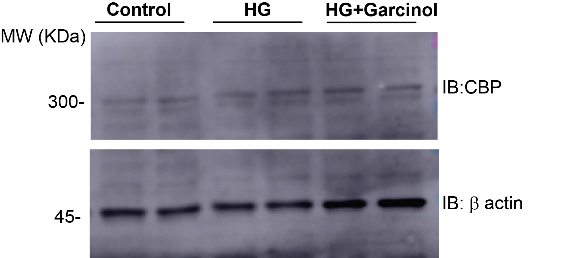


Fig. S7-B. CBP expression in VSMCs treated with HG and garcinol. Human VSMCs (hVSMC) were pre-incubated with 15μM garcinol for 30 min followed by stimulation with 25 mM high glucose (HG) for 12h. Representative blot of CBP expression detected by western blot. β actin used as internal control. Molecular weight (MW).
